# Pushing forward in sarcoma with a new TCR targeting NY-ESO-1

**DOI:** 10.1016/j.xcrm.2023.101159

**Published:** 2023-08-15

**Authors:** Rusul Al-Marayaty, Seth M. Pollack

**Affiliations:** 1 Northwestern University, Feinberg School of Medicine, Department of Medicine, Division of Oncology, Chicago, IL, USA

## Abstract

A phase 1 trial demonstrating the safety and efficacy of a novel NY-ESO-1-specific TCR-T cells by Pan et al.^1^ is a major step forward for adoptive T cell therapy in the clinical practice of advanced soft tissue sarcomas.

## Teaser

A phase 1 trial demonstrating the safety and efficacy of a novel NY-ESO-1-specific TCR-T cells by Pan et al. is a major step forward for adoptive T cell therapy in the clinical practice of advanced soft tissue sarcomas.

## Main text

NY-ESO-1 was first discovered by Lloyd Old and his colleagues as a prototypical Cancer Testis Antigen (CT antigen), a group of immunogenic self-antigens highly expressed in some malignancies and in the testis but only rarely by other healthy tissues in adults.^2^ Its expression in many cancers was characterized by Achim Jungbluth, who found it expressed relatively frequently by most cancers including approximately 20%–30% of melanomas, ovarian cancers, and many others tumor types.^3^ However, Dr. Jungbluth made the important insight that NY-ESO-1 was almost always seen in synovial sarcoma (SS) and usually with a homogeneous pattern of expression.^4^ All of this work required incredible vision and imagination as it happened at a time when modern immunotherapy, including checkpoint inhibitors and engineered cellular therapy, was little more than a dream. Not long afterward, myxoid/round cell liposarcoma (MRCL) was found to have a similar NY-ESO-1 expression pattern.^5^

It did not take long before multiple vaccine trials and cellular therapy trials were targeting NY-ESO-1, including endogenous NY-ESO-1-specific T cells expanded *ex vivo*, high-affinity T cell receptors (TCRs) isolated from patient blood, and notably the National Cancer Institute’s Paul Robbins’ landmark study showing the potential for deep durable responses in individuals with SS and melanoma treated with affinity-enhanced engineered NY-ESO-1-specific T cells enhanced using the alpha-LY TCR.^6^^,^^7^^,^^8^ Since then, MAGE-A4 has joined NY-ESO-1 as an important CT Antigen, asstudies using a TCR for the MAGE-A4_230–239_ epitope, such as the trial led by Hong et al., had similar disease control rates of 64% and objective response rates of 25% and with durable responses tested as part of a large pivotal trial.^9^

In this study, Pan et al. recruited 12 individuals with HLA-A∗02:01 with advanced NY-ESO-1-positive SS (n = 10), MRCL (n = 1), or dedifferentiated liposarcoma (n = 1), designed a novel affinity-enhanced specific TCR-T cell directed against HLA-A∗02:01 NY-ESO-1_157–165_ epitope to explore the adverse effects, created a treatment protocol, and evaluated its efficacy using clinical treatment endpoints and biochemical outcomes.

Treatment included lymphodepleting conditioning with cyclophosamide (45 mg/kg) and fludarabine (60 mg/m^2^) before T cell transfer and low-dose IL-2 (500,000 IU, twice daily for 14 days). Besides the expected bone-marrow-suppression side effects of lymphodepletion, none of the affected individuals experienced treatment-related, serious adverse effects. Cytokine release syndrome is rare for solid tumors, and in sarcoma trials and here, it was seen in 2 of the 12 participants (16.7%) and none of the affected individuals suffered from immune effector cell-associated neurotoxicity syndrome. In this small study, the efficacy seemed promising as five individuals had a partial response while another five had stable disease, culminating to a disease control rate of 83.3% and an objective response rate of 41.7%. Interestingly, both of the individuals with liposarcoma maintained a partial response for over a year following the treatment. When evaluating the persistence and expansion of the infused T cells, genomic copies of the TCR peaked around day 6 after infusion, were found to have a median copy number of >50,000 around that time, and continued to be seen up to 9 months following the infusion. Clinical efficacy was also found to be correlated with the levels of peak values of IFN-γ and CRP, as they were significantly increased in responders than in those who did not respond. Interestingly, higher levels of CXCR3+ cells were associated with a favorable clinical response; this could be linked to the role of IFN-γ in inducing downstream molecules such as CXCR3 expression.

There is currently a crowded landscape for TCR-based therapies in SS and MRCL, with Adaptimmune’s Afami-cel having just completed a large study, and several other TCRs targeting HLA-A0201-restricted epitopes are in development.^10^ The field now desperately needs TCRs targeting epitopes presented by other HLA alleles. TCRs targeted to class II-restricted epitopes may be beneficial. TCRs may also work better in altered microenvironments; SS and MRCL are among the most cold-tumor-immune microenvironments in all of cancer therapy so efficacy of cellular therapy may be improved by agents improving MHC expression and T cell infiltration. Post-treatment therapies, such as IL-15 and other cytokines, may further boost efficacy.

In summary, adoptive T cell therapy targeting CT antigens like NY-ESO-1 is promising for certain patients with advanced SS and MRCL. In this trial, Pan et al. have tested a novel TCR in a treatment protocol with an acceptable adverse effects profile and a durable response rate. Larger phase II trials testing this new TCR are warranted.
